# Clinical and CT findings of COVID-19: differences among three age groups

**DOI:** 10.1186/s12879-020-05154-9

**Published:** 2020-06-22

**Authors:** Jian Wang, Xiandi Zhu, Zhihua Xu, Guangzhao Yang, Guoqun Mao, Yuzhu Jia, Zongyu Xie, Jing Wang, Weiqun Ao

**Affiliations:** 1 Department of Radiology, Tongde Hospital of Zhejiang Province, No. 234, Gucui Road, Hangzhou, 310012 Zhejiang Province China; 2 Department of Radiology, The First Affliated Hospital of Bengbu Medical College, Bengbu, Anhui China; 3 Department of Radiology, XiXi Hospital of Hangzhou, Hangzhou, Zhejiang Province China

**Keywords:** COVID-19, X-ray computed tomography, Age, Pulmonary infection

## Abstract

**Background:**

The novel coronavirus pneumonia (coronavirus disease 2019, COVID-19) has spread around the world. We aimed to recapitulate the clinical and CT imaging features of COVID-19 and their differences in three age groups.

**Methods:**

The clinical and CT data of patients with COVID-19 (n = 307) that had been divided into three groups (Group 1: < 40 years old; Group 2: 40 ≤ age < 60 years old; Group 3: ≥ 60 years old) according to age were analyzed retrospectively.

**Results:**

Of all patients, 114 (37.1%) had histories of epidemiological exposure, 48 (15.6%) were severe/critical cases, 31 had hypertension (10.1%), 15 had diabetes mellitus (4.9%), 3 had chronic obstructive pulmonary disease (COPD, 1%). Among the three groups, severe/critical type, hypertension and diabetes occurred more commonly in the elderly group compared with Group 1&2 (*P* < 0.05, respectively). Cough and chest tightness/pain were more commonly appeared in Group 2&3 compared with Group 1 (*P* < 0.05, respectively). Compared with Group 1 and 2, there were more abnormal laboratory examination indexes (including CRP increase, abnormal percentage of lymphocytes, neutrophils and monocytes) in Group 3 (*P* < 0.05, respectively). CT images revealed that more lobes were affected and more subpleural lesions were involved in the elderly group, besides, crazy paving sign, bronchodilatation and pleural thickening were more commonly seen in the elderly group, with significant difference between Group 1&2, Group 2&3 (*P* < 0.05, respectively).

**Conclusions:**

COVID-19 presented representative clinical manifestations, laboratory examinations and CT findings, but three age groups possessed their own specific characteristics. Grasping the clinical and CT features stratified by age will be helpful for early definite diagnosis of COVID-19.

## Background

At present, the outbreak of the novel coronavirus pneumonia (coronavirus disease 2019, COVID-19) has become a public issue all over the world [1]. By April 24, 2020, more than 2,600,000 people have been diagnosed with COVID-19 [2], and it seems likely that the virus continues to spread. Transmission of COVID-19 through droplets, airborne and physical contact from infected (but not necessarily symptomatic) people, along with the frequent antigenic mutation of the virus make people more easily infected [3–5], and all these factors make it too difficult to control. The incubation period of COVID-19 is usually 2 to 7 days (median 4 days), and can reach up to 24 days [6]. Official WHO data showed that the overall case fatality rate of COVID-19 in China was 5.51% (4642 deaths among 84,311 confirmed cases) [2], which actually might be even lower. Early isolation, early diagnosis, and timely treatment can lower the mortality effectively [6].

The main clinical manifestations of COVID-19 include fever, cough and fatigue. Some patients are presented with stuffy nose, shortness of breath, sore throat and sore muscles [7, 8]. The diagnostic golden standard of COVID-19 is the real-time reverse transcriptase polymerase chain reaction (RT-PCR) test [9], but with low sensitivity [10]. Meanwhile, too many patients are waiting for RT-PCR test due to the insufficient number of nucleic acid detection kits produced by enterprises in such a short time. CT scan is convenient, sensitive and fast, which serves as an important tool for screening, preliminary diagnosis and severity assessment of COVID-19.

Several studies have shown certain specificities of lung CT images in patients with COVID-19 [11, 12], which provided a reliable basis for diagnosis of the disease. Pan [13] et al. summarized CT data of 63 patients, exhibited as patchy consolidation, patchy/punctate ground glass opacities (85.7% of cases), ground glass nodules (22.2%), irregular solid nodules (12.7%) and fibrous stripes (17.5%). However, CT manifestations of COVID-19 are diverse, not all patients’ image performances are typical, pulmonary infection may involve one or more lobes simultaneously, with single, multiple or diffused lesions.

At present, there are few reports about clinical and CT features among different age groups of patients with COVID-19. Whether three age groups possess their own characteristics (including clinical manifestations, laboratory test results and CT features) remains ambiguous. Therefore, the purpose of this study is to analyze the clinical and CT features of patients with COVID-19 in three age groups, so as to better understand the characteristics of COVID-19 and better guide clinical diagnosis and treatment of this infectious disease.

## Methods

### Patients

**Fig. 1:**
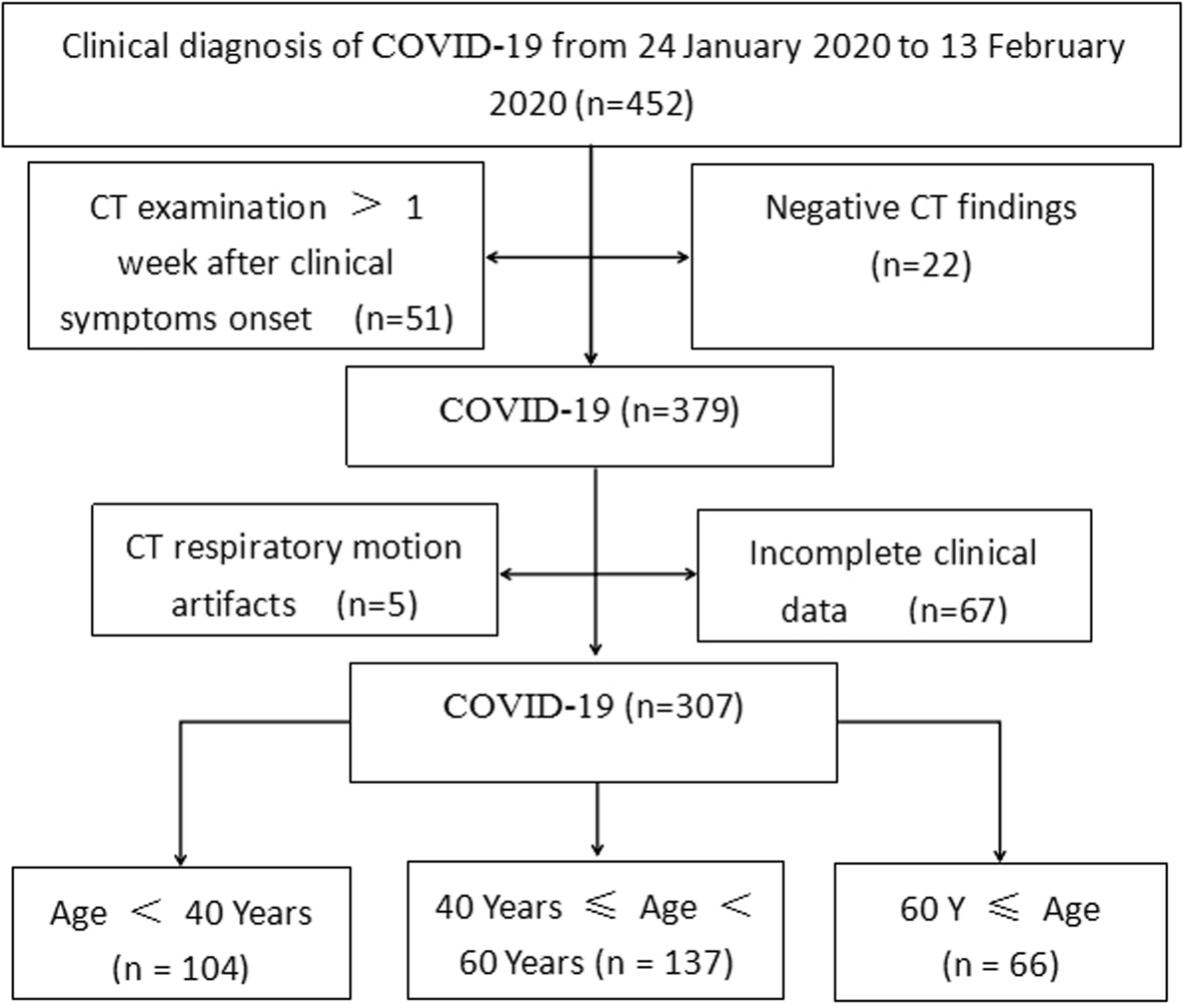
Flow diagram of enrolled patients

This retrospective study was approved by the institutional review board of Tongde Hospital of Zhejiang Province. All patients or their legally authorized representatives had provided written informed consent prior to participation in this study. As a multicenter study, a total of 307 consecutive patients infected with COVID-19 admitted to Tongde Hospital of Zhejiang Province, XiXi Hospital of Hangzhou and the First Affiliated Hospital of Bengbu Medical College were enrolled from January 24, 2020 to February 23, 2020. As teaching hospitals with more than 2000 beds, all of the three hospitals treated patients independently. This study included a small portion of the patient data from our previous research [14]. The Flow chart showed the enrollment procedure of patients with COVID-19. The inclusion criterion was that patients were positive for RT-PCR test of COVID-19. For those suspected cases, we performed three to seven times of RT-PCR tests to reduce false negative diagnosis. To sum up, 307 patients were enrolled in this study (Fig. 1), of whom 202 (65.8%) was positive for the first RT-PCR test of COVID-19, 72 (23.5%) was positive for the second test, 27 (8.8%) was positive for the third test and 6 (1.9%) turned positive with four to seven times of detection. Due to rapid changes in CT manifestations of COVID-19 after treatment, CT examination was done at ≤1 week after clinical symptoms onset. Their baseline clinical and image data were reviewed retrospectively.

### Clinical information

**Fig. 2:**
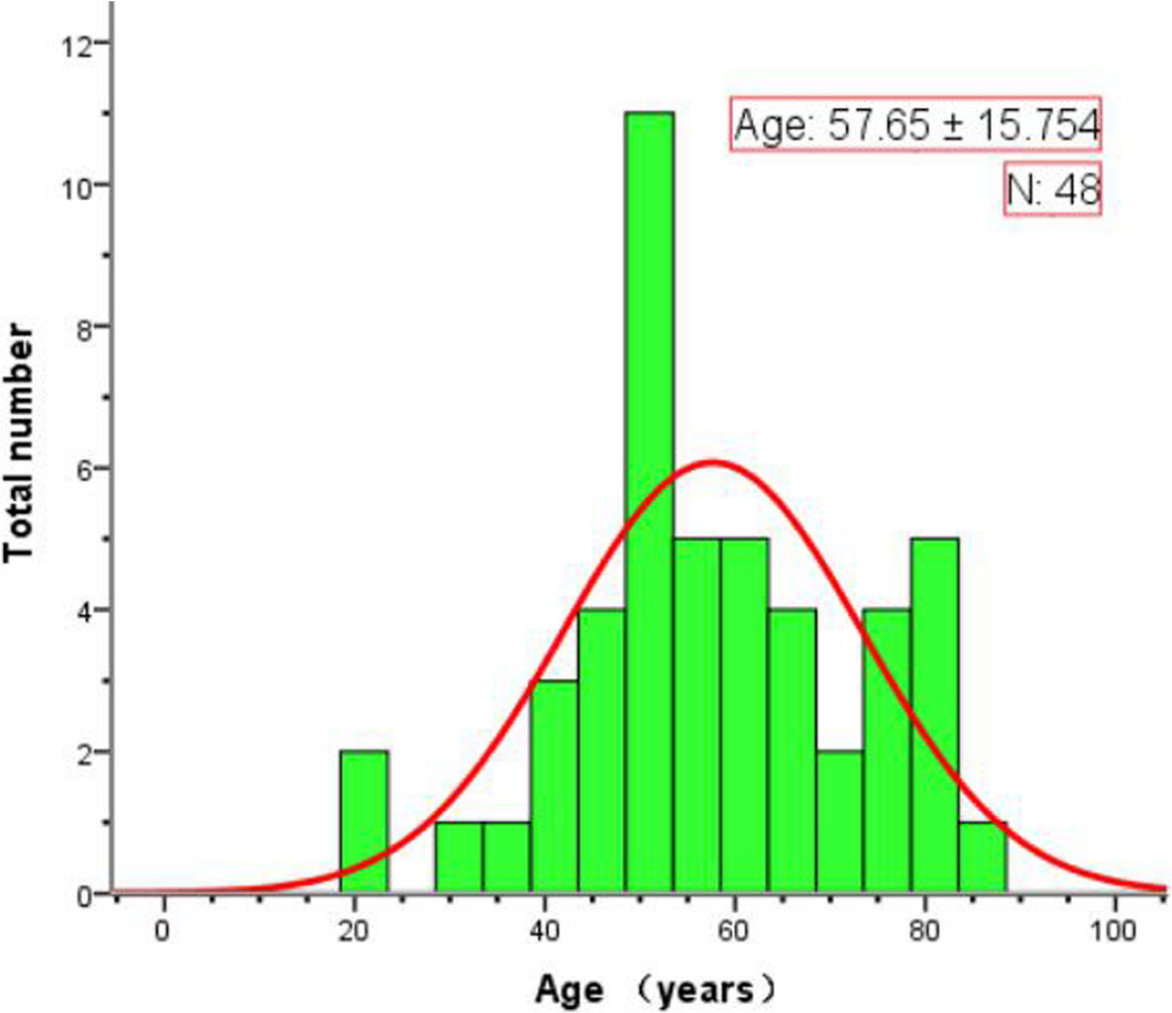
Stacked bars (green) show the age and number distribution of the heavy/critical type of COVID-19. The curve (red) represents the number distribution trend of the heavy/critical type according to the levels of age

Baseline information includes: gender, age (divide into three groups: Group 1: < 40 years old; Group 2: 40 ≤ age < 60 years old; Group 3: ≥ 60 years old), history of epidemiological exposure (a history of traveling to or residence in Wuhan and adjacent cities, China or communities with local cases in the last 14 days before symptom onset; close contact with patients with fever or respiratory symptoms from Wuhan and adjacent cities, China or communities with local cases in the last 14 days before symptom onset; epidemiologically connected to COVID-19 infections or clustered onsets), clinical symptoms (presence of fever, body temperature, cough, expectoration, fatigue, chest tightness, breathlessness and others) and laboratory examinations [C-reactive protein (CRP), white blood cell (WBC), lymphocyte, neutrophil, monocyte] of all patients were collected and evaluated. According to the National Recommendations for Diagnosis and Treatment of Respiratory Infections Caused by COVID-19 (the 6th Edition, released on Feb 19, 2020) [15], the patients were classified into: mild/moderate type versus severe/critical type. The age and number distribution of the severe/critical type was shown in Fig. 2.

### Imaging protocol and analysis

All patients underwent chest 16-MDCT (SOMATOM Emotion16, SIEMENS, Germany), 64-MDCT (Definition AS, SIEMENS, Germany) or 64-MDCT (Optima CT680, GE, USA) scans. CT scanning parameters were as follows: display field of view (dFOV) 32 ~ 35 cm, tube voltage 120 ~ 130 kV; tube current 35 ~ 50 mA, slice thickness 5 mm. All CT images were reconstructed with the slice thickness of 1.25 mm. The CT images were independently reviewed on the Picture Archiving and Communication System (ADW4.6, GE, USA) by two cardiothoracic radiologists (J.W. and W.A.) who had 14-year (J.W.) and 13-year (W.A.) experience in interpreting cardiothoracic images, respectively. Disagreements were resolved by consensus. Kappa statistic was also employed to evaluate the consistency of CT features between two radiologists. All CT images were assessed on pulmonary window and mediastinal window for the following features: (1) presence of GGO (ground-glass opacity) or/and consolidation; (2) nodules or/and fibrosis; (3) number of pulmonary lobes involved; (4) number and distribution of lesions; (5) presence of pleural effusion or/and pleural thickening; (6) presence of bronchodilatation or/and vascular dilatation; (7) Special imaging signs such as crazy paving sign, cavity sign, halo sign, reversed halo sign; (8) lymphadenectasis.

**Fig. 3:**
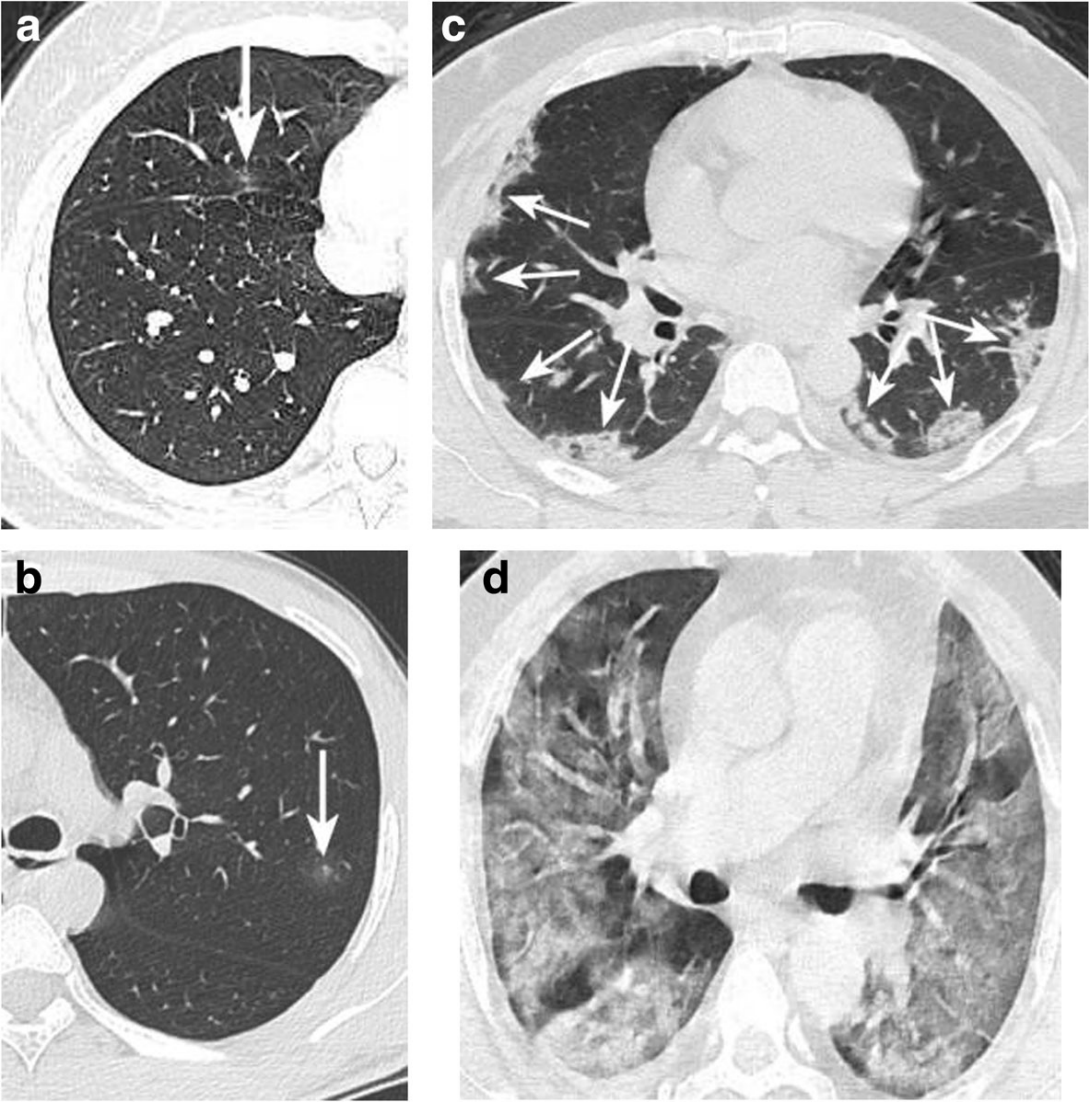
Atypical case **a**: Mild type patient. CT showed a small pGGO in the right middle lobe (arrow), and no abnormality in the remaining lobes. Atypical case **b**: Mild type patient. Only a small pGGO was detected in the left upper lobe with hale sign (arrow). Typical case **c**: Moderate type patient. CT showed multiple GGOs in the bilateral lobes with vascular dilatation and halo sign (arrow). Typical case **d**: Heavy type patient. CT showed diffused GGOs in the bilateral lobes

**Fig. 4:**
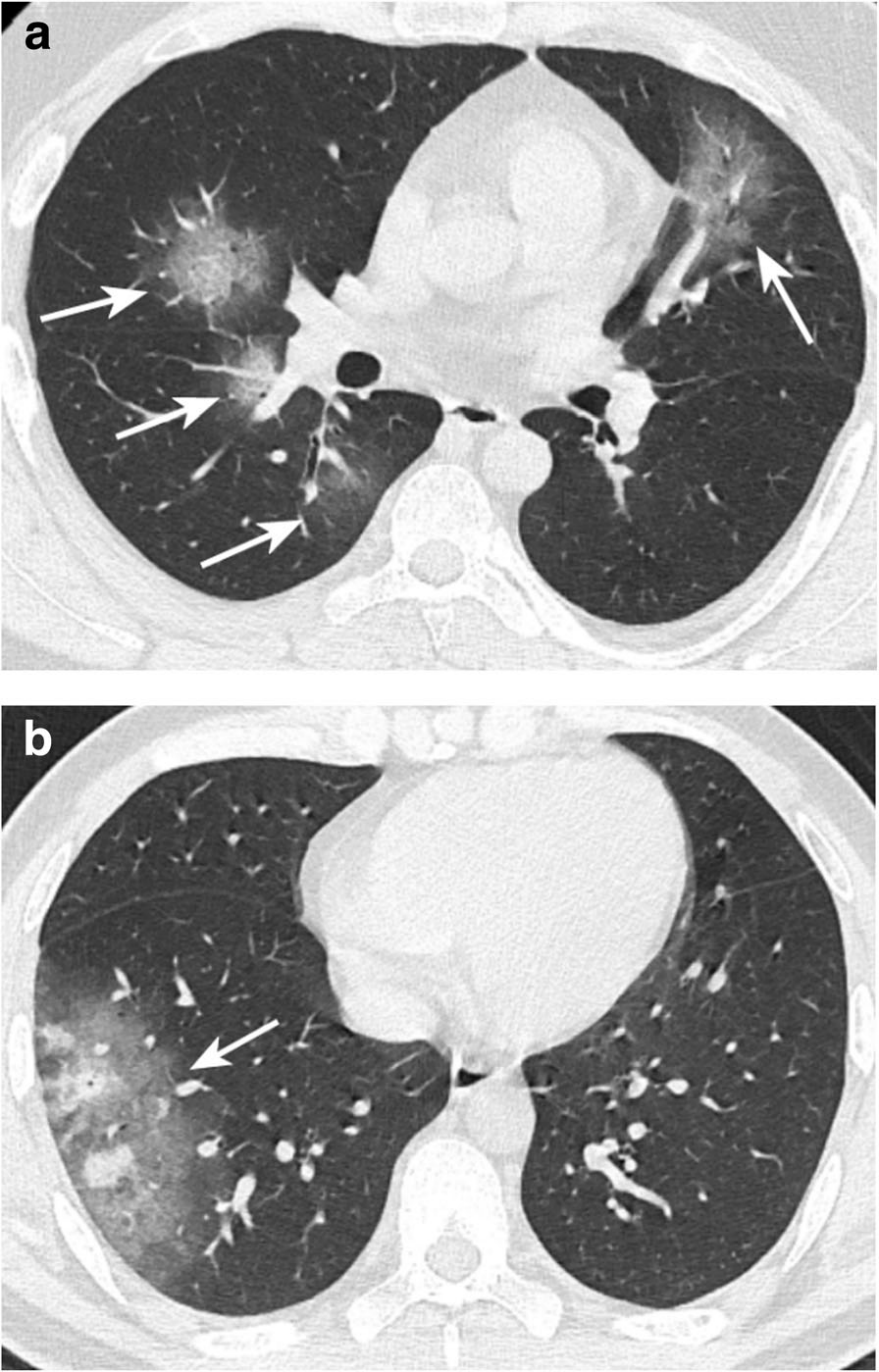
Typical pGGO case **a**: Moderate type patient. CT showed multiple pGGOs in the bilateral lung lobes with halo sign (arrow). Some of the lesions were located under the pleura. Typical mGGO case **b**: Moderate type patient. CT showed a subpleural mGGO within a solitary nodule (arrow)

**Fig. 5:**
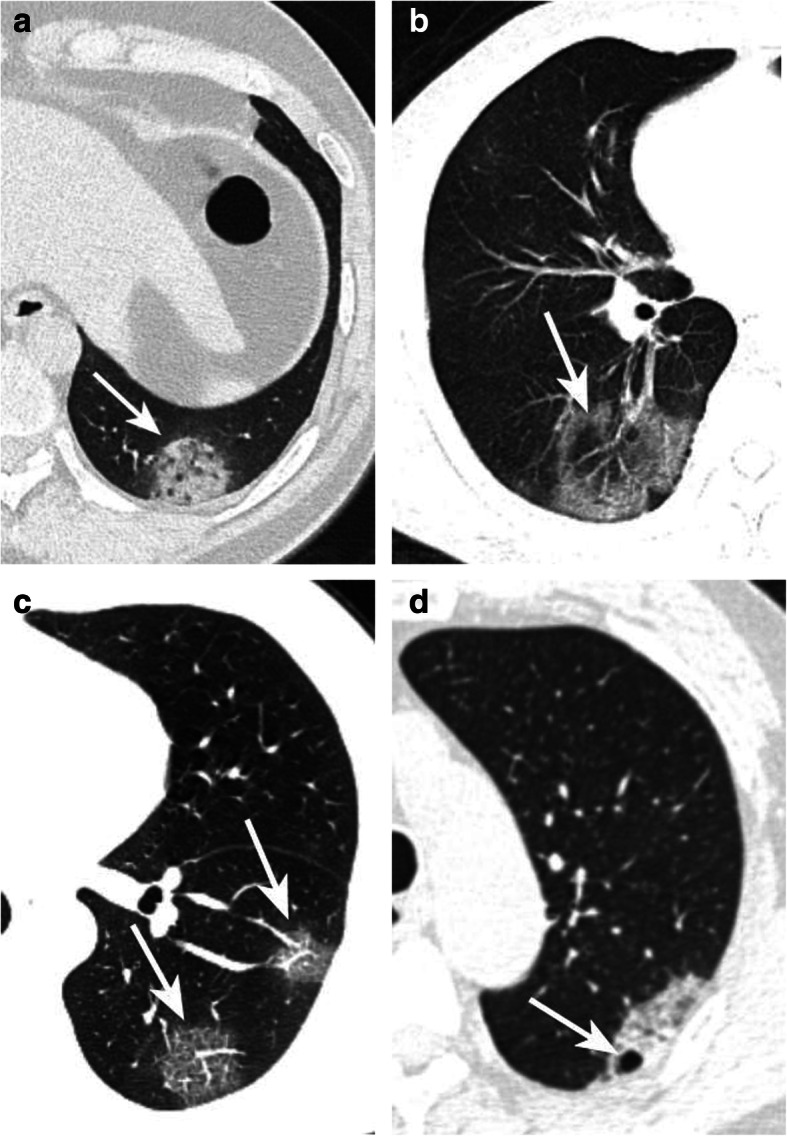
Typical CT signs. Halo sign **a**: Moderate type patient. CT showed a pGGO with halo sign in the left low lobe (arrow). reversed halo sign **b**: Moderate type patient. CT showed a mGGO with reversed halo sign (arrow). Crazy paving sign **c**: Moderate type patient. CT showed two pGGOs with crazy paving sign in the left low lobe (arrow). Cavity sign **d**: Moderate type patient. CT showed a pGGO with a small cavity (arrow)

GGO: subtle ground glass opacities that are seen around the vessel and small airways (Fig. 3 a). Halo sign: GGO around the tumor or nodule (Fig. 4 a). reversed halo sign: presence of central GGO surrounded by the air-space consolidation of crescentic or ring shape (Fig. 5 b). Crazy paving sign: presence of GGO with interlobular septa thickening (Fig. 5 c). Cavity sign: gas accumulation in a space, manifested as a low-density or transparent region in the pulmonary lesions (Fig. 5 d).

### Statistical analysis

The data were analyzed using the Statistical Package for Social Sciences for Windows, Version 20 (IBM Corp, Armonk, NY, USA). Categorical variables were described as frequencies and percentages. Normally distributed continuous variables were described as means and standard deviations (SD), and non-parametric data were described as medians and interquartile ranges. Chi-square test was used for all categorical variables in the three groups. Statistical significance was defined as *P* < 0.05. Kappa statistic was employed to evaluate the consistency of CT features between the two radiologists.

## Results

### General data analysis

The clinical and CT data of 307 patients with COVID-19 were analyzed. Basic information: 156 cases (50.8%) were male, 151 cases (49.2%) were female, the average age was 46.2 ± 17.11 years old. RT-PCR tests of COVID-19 were positive for all patients, with 114 patients (37.1%) had histories of epidemiological exposure. Of all patients, 259 (84.6%) were categorized into mild/moderate type, 48 (15.6%) severe/critical type. 31(10.1%) had hypertension, 15 (4.9%) had diabetes mellitus, 8 (2.6%) had Hepatitis B and 3 (1%) had COPD. Patients generally had hypertension, diabetes or COPD in Group 3. Clinical symptoms were as follows: fever (301 cases, 98%), cough (239 cases, 77.6%), fatigue (51 cases, 16.6%), sore throat (37 cases, 12.1%), chest tightness or pain (34 cases, 11.1%), dizziness or pain (28 cases, 9.1%), aching muscles (27 cases, 8.8%), hyperpnea (21 cases, 6.8%) and nasal stuffiness (20 cases, 6.5%). Laboratory examinations: CRP increased in 182 cases (59.3%), abnormal lymphocyte, neutrophil, monocyte and WBC counts in 148 (48.2%), 95 (30.9%), 70 (22.8%) and 60 (19.5%) cases, respectively (Table 1).

**Table 1:** Clinical data characteristics of three age groups of COVID-19 patients

Groups	All patients (*n* = 307)	Age < 40 Y (*n* = 104)	40 Y ≤ Age <60 Y (*n* = 137)	60 Y ≤ Age (*n* = 66)	*P1*(G1 & G2)	*P2*(G1 &G3)	*P3*(G2 &G3)
CBI							
Sex (male, %)	156 (50.8%)	53 (51%)	72 (52.6%)	31 (47%)	0.806	0.612	0.456
Age (M ± SD, Y) (Rang, Y)	46.2 ± 17.11 (1 ~ 89)	2.3 ± 8.35 (1 ~ 39)	49.3 ± 5.35 (40 ~ 59)	69.3 ± 8.51 l(60 ~ 89)	/	/	/
HD							
Hypertension	31 (10.1%)	0	16 (11.7%)	15 (22.7%)	<0.001	<0.001	0.040
Diabetes	15 (4.9%)	0	9 (6.6%)	6 (9.1%)	0.020	0.007	0.721
Hepatitis B	8 (2.6%)	1 (1%)	7 (5.1%)	0	0.156	1.000	0.145
COPD	3 (1%)	0	0	3 (4.5%)	1.000	0.058	0.110
Clinical symptoms							
Fever	301 (98%)	100 (96.2%)	135 (98.5%)	66 (100%)	0.447	0.274	0.820
Cough	239 (77.9%)	79 (76%)	101 (73.7%)	59 (89.4%)	0.692	0.029	0.010
Fatigue	51 (16.6%)	13 (12.5%)	23 (16.8%)	15 (22.7%)	0.355	0.080	0.310
Sore throat	37 (12.1%)	14 (13.5%)	14 (10.2%)	9 (13.6%)	0.453	0.955	0.472
Chest tightness or pain	34 (11.1%)	6 (5.8%)	12 (8.8%)	16 (24.2%)	0.382	<0.001	0.003
Dizziness or pain	28 (9.1%)	8 (7.7%)	13 (9.5%)	7 (10.6%)	0.624	0.514	0.802
Aching muscles	27 (8.8%)	5 (4.8%)	15 (10.9%)	7 (10.6%)	0.087	0.258	0.941
Hyperpnea	21 (6.8%)	3 (2.9%)	10 (7.3%)	8 (12.1%)	0.133	0.039	0.258
Nasal stuffiness	20 (6.5)	8 (7.7%)	11 (8%)	1 (1.5%)	0.923	0.161	0.127
Others#	8 (2.6%)	2 (1.9%)	3 (2.2%))	3 (4.5%)	1.000	0.603	0.627
LE							
CRP increase	182 (59.3%)	47 (45.2%)	93 (67.9%)	42 (63.6%)	<0.001	0.019	0.548
LY% abnormal	148 (48.2%)	35 (33.7%)6↑	71 (51.8%)1↑	42 (63.6%)	0.005	<0.001	0.113
NEUT% abnormal	95 (30.9%)	21 (20.2%)5↓	43 (31.4%)3↓	31 (47%)	0.051	<0.001	0.031
MO% abnormal	70 (22.8%)	31 (29.8%)2↓	33 (24.1%)	6 (9.1%)	0.319	0.001	0.011
WBC% abnormal	60 (19.5%)	19 (18.3%)2↑	21 (15.3%)4↑	20 (30.3%)9↑	0.543	0.069	0.013
Clinical types					0.002	<0.001	0.015
Mild/Moderate	259 (84.6%)	100 (96.2%)	114 (83.2%)	45 (68.2%)			
Severe/Critical	48 (15.6%)	4 (3.8%)	23 (16.8%)	21 (31.8%)			
HEE	114 (37.1%)	49 (47.1%)	56 (40.9%)	9 (13.6%)	0.333	<0.001	<0.001

Note: *Y* years, *G1* Age<40 Y, *G2* 40 Y ≤ Age<60 Y, *G3* 60 Y ≤ Age, *CBI* Clinical baseline information, *COPD* chronic obstructive pulmonary disease, *HD* History of disease, *LE* laboratory examination; CRP: C-reactive protein (normal: 0 ~ 10 mg/); WBC: white blood cell (normal: 3.5 ~ 9.5 × 10^9^), *Ly* lymphocyte (normal: 20% ~ 40%), *Mo* monocyte (normal: 3 ~ 10%), *NEUT* neutrophil (normal: 40% ~ 75%), *HEE* history of epidemiological exposure, Others* Major diseases or surgical history, *Others#* diarrhoea or vomiting

CT findings of COVID-19 were as follows (Table 2): 85 (27.7%) patients had one or two affected lobes, 222 (72.3%) had multi-affected lobes. Subpleural lesions appeared in 289 (94.1%) patients. pGGO was detected in 276 (89.9%) cases, mGGO (191 cases, 62.2%), halo sign (254 cases, 82.7%), crazy paving sign (117 cases, 38.1%), vascular dilatation (249 cases, 81.1%), bronchial dilatation (146 cases, 47.6%). Other less common CT manifestations include reversed halo sign (38 cases,12.4%), cavity sign (21 cases, 6.8%), pleural thickening (36 cases, 11.7%), pleural effusion (15 cases, 4.9%) and lymphadenectasis (13 cases, 4.2%).

**Table 2:** CT characteristics of three age groups of COVID-19 patients

Groups	All patients (*n* = 307)	Age < 40 Y (*n* = 104)	40 Y ≤ Age <60 Y (*n* = 137)	60 Y ≤ Age (*n* = 66)	*P1*(G1 & G2)	*P2*(G1 &G3)	*P3*(G2 &G3)
CT Findings (LW)							
Location					<0.001	<0.001	0.240
One lobe or Two lobes	85 (27.7%)	58 (55.8%)	28 (20.4%)	9 (13.6%)			
Multiple lobes	222 (72.3%)	46 (44.2%)	109 (79.6%)	57 (86.4%)			
Number					<0.001	<0.001	1.000
One or two	31 (10.1%)	23 (22.1%)	5 (3.6%)	3 (4.5%)			
More	276 (89.9%)	81 (77.9%)	132 (96.4%)	63 (95.5%)			
Subpleural lesions	289 (94.1%)	90 (86.5%)	135 (98.5%)	64 (97%)	<0.001	0.023	0.830
Shape							
pGGO	276 (89.9%)	99 (95.2%)	123 (89.8%)	54 (81.8%)	0.123	0.005	0.112
mGGO	191 (62.2%)	60 (57.7%)	85 (62%)	46 (69.7%)	0.494	0.115	0.286
Sign							
Halo sign	254 (82.7%)	86 (82.7%)	114 (83.2%)	54 (81.8%)	0.915	0.884	0.806
Reversed halo sign	38 (12.4%)	8 (7.7%)	22 (16.1%)	8 (12.1%)	0.051	0.335	0.459
Crazy paving sign	117 (38.1%)	18 (17.3%)	66 (48.2%)	33 (50%)	<0.001	<0.001	0.808
Cavity sign	21 (6.8%)	3 (2.9%)	11 (8.3%))	8 (12.1%)	0.091	0.039	0.348
Organizational involvement							
Vascular dilatation	249 (81.1%)	77 (74%)	112 (81.8%)	60 (90.9%)	0.053	0.002	0.089
Bronchodilatation	146 (47.6%)	38 (36.5%)	69 (50.4%)	39 (59.1%)	0.032	0.004	0.243
CT Findings (MW)							
Pleural thickening*	36 (11.7%)	4 (3.8%)	21 (15.3%)	11 (16.7%)	0.004	0.004	0.806
Pleural effusion*	15 (4.9%)	4 (3.8%)	6 (4.4%)	5 (7.6%)	1.000	0.480	0.541
Lymphadenectasis	13 (4.2%)	3 (2.9%)	5 (3.6%)	5 (7.6%)	1.000	0.300	0.144

Note: *G1* Age<40 Y, *G2* 40 Y ≤ Age<60 Y, *G3* 60 Y ≤ Age, *LW* lung window, *MW* mediastinal window, *pGGO* pure ground-glass opacity, *mGGO* mixed ground-glass opacity, * All but one patients had mild pleural thickening or a little effusion (the only one case had moderate pleural effusion)

### Clinical characteristics of three age groups

Clinical data analysis of COVID-19 for three age groups was shown in Table 1. Severe/Critical type, high blood pressure and diabetes mellitus were more commonly occurred in the elderly group compared with Group 1&2 (*P* < 0.05, respectively). Cough and Chest tightness/pain were more commonly appeared in Group 2&3 compared with Group 1 (*P* < 0.05, respectively). CRP increase and lymphocyte count abnormality were significantly different between Group 1&2, Group 1&3 (*P* < 0.05, respectively), while abnormal neutrophil and monocyte counts were significantly different between Group 1&3, Group 2&3 (*P* < 0.05, respectively). The difference of abnormal WBC count was significant between Group 2&3 (*P* < 0.05) as well. Of all Severe/Critical type patients, 18 had hypertension (37.5%), 11 had diabetes (22.9%), 3 had hepatitis B (6.3%), and 3 had COPD (6.3%), which were much higher than that of the Mild/ Moderate type. Compared with patients who had no basic diseases, patients with basic diseases (Hypertension, Diabetes, hepatitis B and COPD) are more likely to be Severe/Critical type (*P* < 0.05) (Table 3).

**Table 3:** Relationship with the severity of COVID-19 and underlying diseases

UD* Clinical types	Hypertension *n* = 31	Diabetes *n* = 15	Hepatitis B *n* = 8	COPD *n* = 3	NUD *n* = 263	*P* 1	*P2*	*P3*	*P4*
Mild/Moderate	13	4	5	0	243	<0.001	<0.001	0.019	<0.001
Severe/Critical	18	11	3	3	20				

UD* underlying diseases, patients with 1–3 basic diseases, *NUD* No underlying diseases, *P1* Hypertension & NUD, *P2* Diabetes & NUD, *P3* Hepatitis B & NUD, *P4* COPD & NUD

### Comparative analysis of CT image characteristics among three age groups

**Fig. 6:**
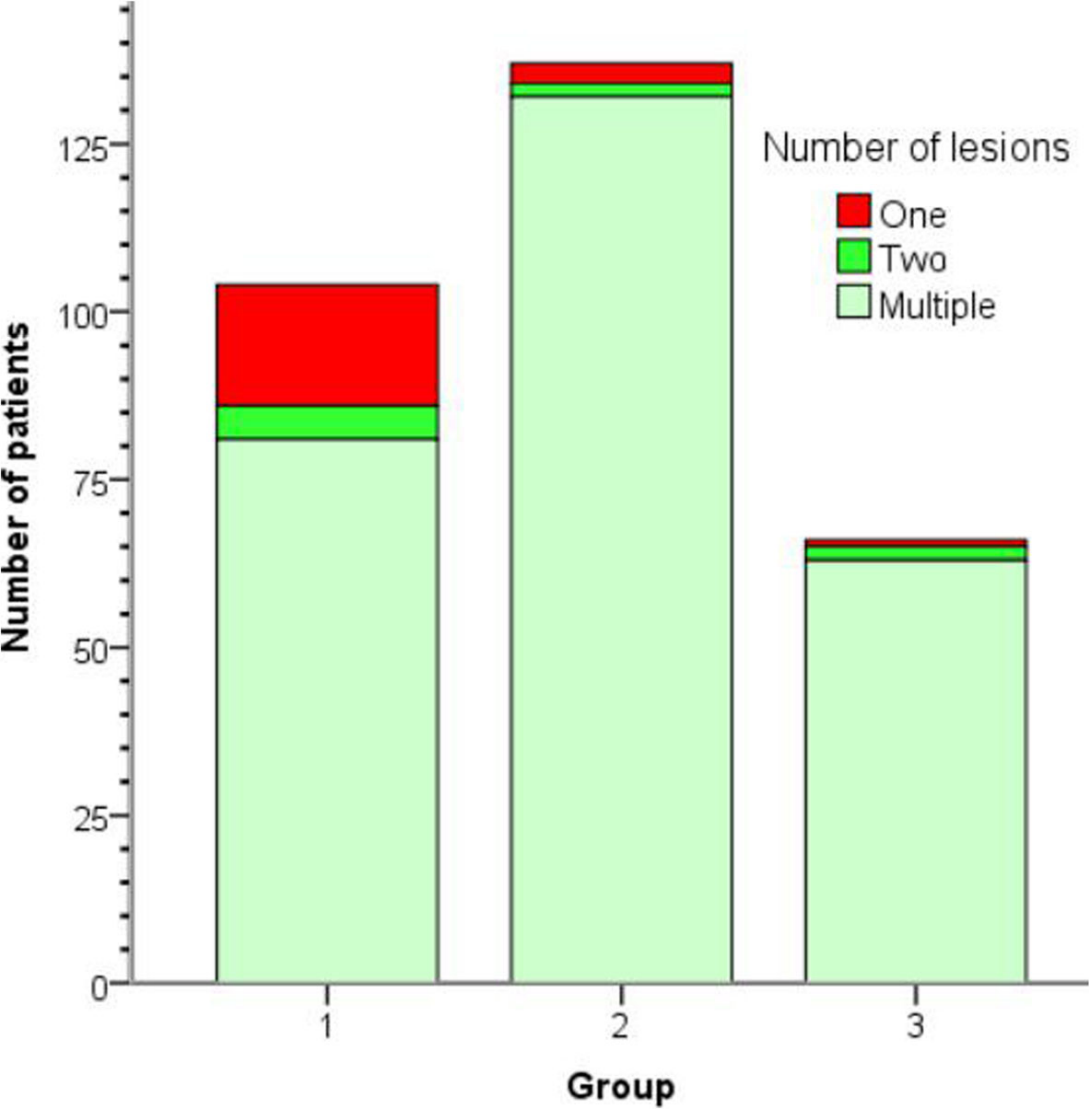
Stacked bars show the distribution of number of lesions on CT images for three age groups. Patients were grouped by age: (Group 1: < 40 years old; *n* = 104), (Group 2:40 ≤ age < 60 years old; *n* = 137), (Group 3: ≥ 60 years old; *n* = 66)

CT feature analysis of COVID-19 for three age groups was described in Table 2. More number of affected lobes, lesions and subpleural involved lesions were detected in the elderly group, these differences were significant between Group 1&2, Group 2&3 (*P* < 0.01, respectively). Moreover, crazy paving sign, bronchodilatation and pleural thickening were more commonly presented in the elderly group, the differences were significant between Group 1&2, group 2&3 (*P* < 0.05, respectively). Besides, the number of pGGO, crazy paving sign and vascular dilatation between Group 1&3 were statistically different (*P* < 0.05, respectively). Distribution of the number of lesions on CT images at three age groups was shown in Fig. 6. There was a good consistency between the two radiologists in evaluation of CT features of COVID-19 (kappa values ranged from 0.66 to 0.81).

## Discussion

Of all patients, 114 out of 307 (37.1%) had a history of epidemiological exposure. Fever, cough and fatigue are typical clinical manifestations of COVID-19. Other clinical manifestations include sore throat, chest tightness or pain, dizziness, aching muscles, hyperpnea and nasal stuffiness. Wang et al. [16] reported common symptoms of 138 patients were fever (98.6%), cough (59.4%) and fatigue (69.6%), which is similar to our study. The rest clinical manifestations resemble those previously reported [16–20]. It is worth mentioning that higher incidence of severe/critical type of COVID-19 was detected in the elderly group, which might be associated with advanced age and coexistence of basic diseases (hypertension and diabetes). In addition, cough and chest tightness/pain were more commonly emerged in Group 3, which was similar to previous reports [5, 21].

In our study, among all laboratory examinations, CRP increase was the most frequent (59.3%), followed by lymphocytes decrease, then abnormality of neutrophil and WBC counts. It is worth pointing out that few young patients got elevated lymphocytes, which might be ascribed to their strong immune system. Furthermore, nearly half of the elderly patients had mildly elevated WBC, while it was rare in the younger group, such phenomenon was similar to a previously reported study [22]. These results have great value in assessment of COVID-19. In particular, decreased WBC has an important differential diagnosis value when compared with common pneumonia, which is usually accompanied by increased WBC. Compared with Group 1 and 2, there are more abnormal laboratory examination indexes (including CRP increase, abnormal number of lymphocytes, neutrophils and monocytes) in Group 3. It may be related to underlying diseases and immunological dysfunction in aged patients. By summarizing 99 cases, Chen et al. [23] found that COVID-19 was more likely to affect elderly men with comorbidities, and could result in acute respiratory distress syndrome (ARDS). Another study [7] had shown that older patients were correlated with higher severity and mortality of COVID-19, which they found the median age of death was 75 years old for COVID-19, and the median time from symptom onset to death in patients aged 70 and above (11.5 days) was shorter than those below 70 years old (20 days). These results demonstrated that the disease might progress faster in the older patients than in the young.

It is noteworthy that compared with patients who had no basic diseases, patients with basic diseases (Hypertension, Diabetes, hepatitis B and COPD) are more likely to develop into Severe/Critical type. Human pathogenic coronaviruses gain entry into their target cells through angiotensin-converting enzyme 2 (ACE2), which is expressed by epithelial cells of the lung, kidney, vessels and intestine. The increased expression of ACE2 would facilitate infection of COVID-19 [24]. For patients with diabetes or hypertension, treatment with ACE2-increasing drugs increases the risk for severe and fatal COVID-19 infection [25]. A recent study showed that the occurrence of COPD was associated with a nearly four-fold higher risk of developing severe COVID-19 [24]. COPD is defined as chronic infection of large (central) airway, small (peripheral) bronchioles and damage of lung parenchyma. Viral infections caused acute exacerbation of COPD, which can result in impairment of lung function in many patients [26]. A large cohort including 1099 COVID-19 cases indicated that 21 (2.1%) had hepatitis B, more critical type patients had abnormal liver aminotransferase levels than mild type patients [6]. The study suggested that patients with chronic hepatitis were associated with higher mortality after infection of COVID-19 [6]. Therefore, we suggest that basic diseases are crucial risk factors for severe/critical type of COVID-19.

Our study showed COVID-19 had its own representative imaging manifestations. Patients might have one affected lobe or multiple affected lobes. Typical image presentations of COVID-19 include multiple patchy/punctate pGGO or mGGO mainly distributed in subpleural areas, accompanied by halo sign, crazy paving sign, vascular and bronchial dilatation, which were consistent with literature reports [27, 28]. The virus was likely to attack peripheral vascular and bronchus in the early stage of the disease, which caused the increase of the intraductal pressure and resulted in exudation, as reflected by the subpleural pGGO and halo sign. Over time, crazy paving sign was formed due to thickening of the interlobular septum and increased exudation of the alveolus. If the disease continued to progress, the thickened lobular septum limited the absorption of the alveolar exudation, resulting in the alveolar consolidation and mGGO formation. Other less common CT manifestations include reversed halo sign, cavity sign, mild pleural thickening, a little pleural effusion and lymphadenectasis, whose incidence were similar to previous reports [29, 30]. Generally, non-subpleural distribution, single lesion and 1 or 2 affected lobes were more common in Group 1. However, compared with Group 1, multiple lesions, more affected lobes and a wider range of infections were involved in Group 2 and 3. When the ventilation function was seriously impaired, lung CT performance could progress to a “white lung” appearance [31], which was more commonly detected in old patients than young patients. Song et al. [11] found by investigation of 51 patients with COVID-19 that younger patients tended to have more GGOs, while elderly patients tended to have more consolidations and more involved areas of lung. We think it may serve as an alert in the management of patients by identifying these age-related CT signs of COVID-19.

Our study revealed that mild pleural thickening was more commonly seen in elderly patients, most pulmonary lesions existed in multiple lobes, with predominant distribution in posterior and peripheral parts of the lung that were always tightly close to the pleura. Pleural thickening often indicated that the lesion of COVID-19 has invaded the bronchioles and alveolar epithelium of the cortical lung tissue, and the distribution of lesions gradually expanded from the periphery to the central part of the lung. We also found that bronchiectasis was more commonly seen in elderly patients than young patients. In addition, we have noticed that many elderly patients had a history of bronchiectasis. Bronchiectasis may be one of the risk factors for severe/critical type of COVID-19, which needs further study.

It is worth noting that lesions of 18 cases (with 14 in Group 1) were not in the subpleural areas, such imaging features were atypical and might easily be mistaken for common inflammatory GGO. For atypical CT findings, we should pay high attention to timely insulate the suspected cases to avoid possible transmission, meanwhile review the epidemiological histories and suggest RT-PCR test and follow-up CT scans, since most positive cases have a faster progression of CT manifestations in a short time.

Due to the responsible viruses are also coronaviruses, CT manifestations of COVID-19 are similar to those reported with MERS and SARS. CT features such as GGO, consolidation and crazy paving sign were also seen in MERS and SARS. Likewise, pleural effusion and pneumothorax were commonly seen in critical patients with previous Coronavirus pneumonias [32]. In our study, multiple lobes (72.3%) involved by GGO or consolidation (85.7%) distributed in subpleural areas (94.1%) were the main CT manifestations of COVID-19. Moreover, other CT signs such as halo sign, reversed halo sign, crazy paving sign, cavity, vascular dilatation and pleural thickening were commonly seen in our study. To our knowledge, pulmonary cavitation and reversed halo sign have not been mentioned in the literatures about MERS and SARS. Furthermore, unifocal involvement is more prevalent than multifocal involvement on CT of patients with previous Coronavirus pneumonias. As the infectious diseases of MERS or SARS progress, pulmonary infection focus eventually spread to the central area and bilateral upper lobes [33], which is different from COVID-19 in our study.

There are some limitations in this study: 1) We have not evaluated dynamic imaging progress of COVID-19. 2) This study mostly emphasized on the CT imaging features of COVID-19 and the differences among three age groups, yet which CT findings could be used for directing treatment and estimating prognosis of COVID-19 had not been investigated, which will be the concern of the follow-up study.

## Conclusion

In summary, our study suggested that elderly age coexisted with basic diseases might serve as important risk factors for heavy/critical type of COVID-19. Compared with young patients, there are more abnormal laboratory examination indexes in elderly patients. CT images revealed that multi-affected lobes, subpleural lesions, crazy paving sign, bronchodilatation and pleural thickening were more commonly seen in the elderly patients than young patients. In some young patients with mild/moderate type, GGO may not appear in the subpleural areas. These atypical CT features need further attention and follow-up is important. Therefore, three age groups of COVID-19 possess their own characteristics. Grasping these characteristics will be helpful for the clinicians to better understand the characteristics of COVID-19 and better guide clinical diagnosis and treatment of this infectious disease.

## Data Availability

The datasets used and/or analyzed during the current study are available from the corresponding author on reasonable request.

## Abbreviations

ACE2: Angiotensin-converting enzyme 2
CRP: C-reactive protein
COPD: Chronic obstructive pulmonary disease
GGO: Ground ground-glass opacity
mGGO: Mixed ground-glass opacity
MERS: Middle East Respiratory Syndrome
pGGO: Pure ground-glass opacity
RT-PCR: Real-time reverse transcriptase polymerase chain reaction
SARS: Severe acute respiratory syndrome
WBC: White blood cell
